# Supplementary material for: Mitochondrial Aldehyde Dehydrogenase 2 (ALDH2) Protects against Binge Alcohol-Mediated Gut and Brain Injury
Source: Cells. 2024 May 28;13(11):927. doi: 10.3390/cells13110927 (PMC11171926; doi:10.3390/cells13110927)
Supplement: Supplementary file 1 [file cells-13-00927-s001.zip › cells-2990553-supplementary (1).pdf]

Supplementary Information

Supplementary Figure S1

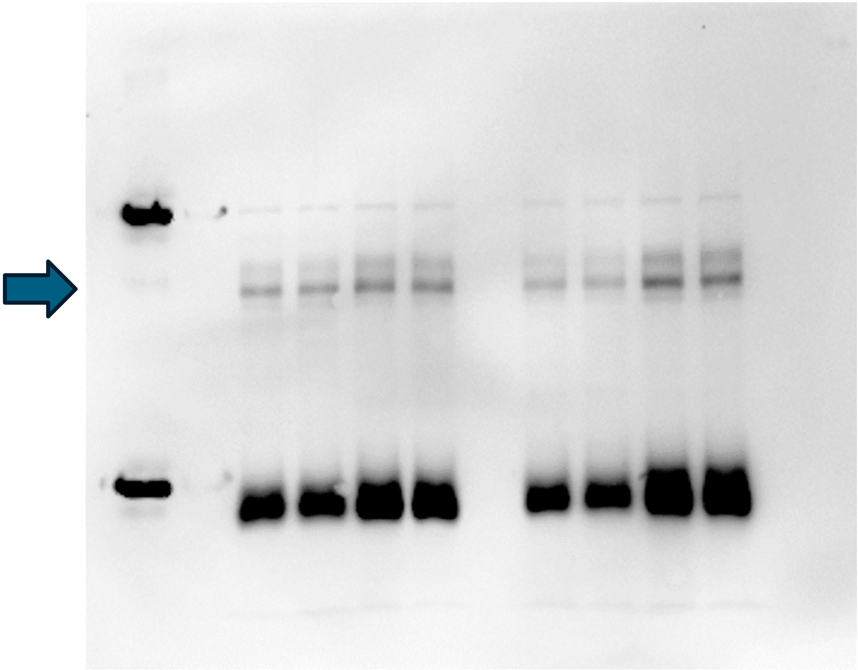

Immunoblot analysis of gut 3-NT (Fig. 3A)

Supplementary Figure S2

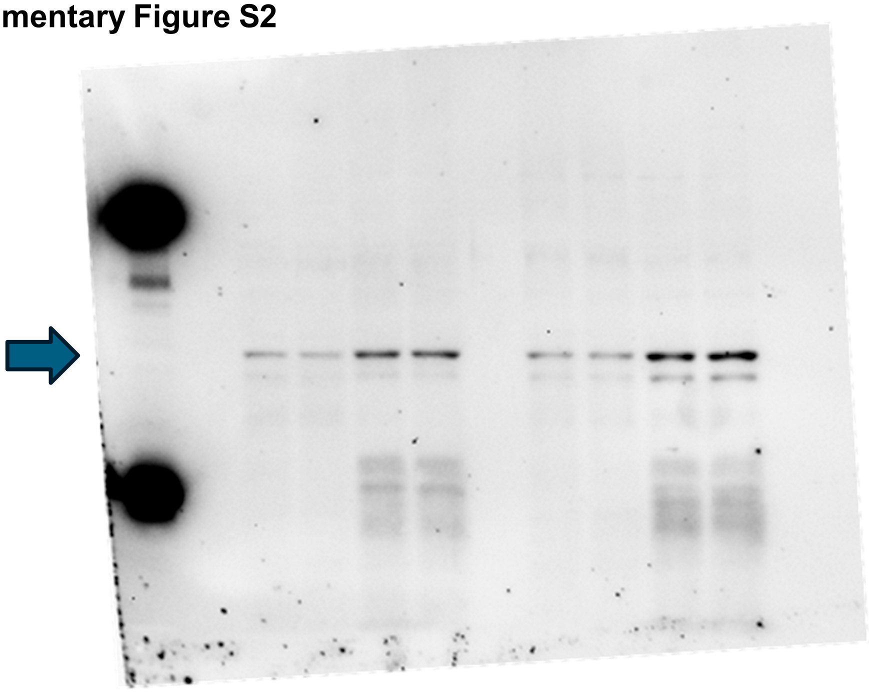

Immunoblot analysis of gut Ac-Lys (Fig. 3A)

Supplementary Information

Supplementary Figure S3

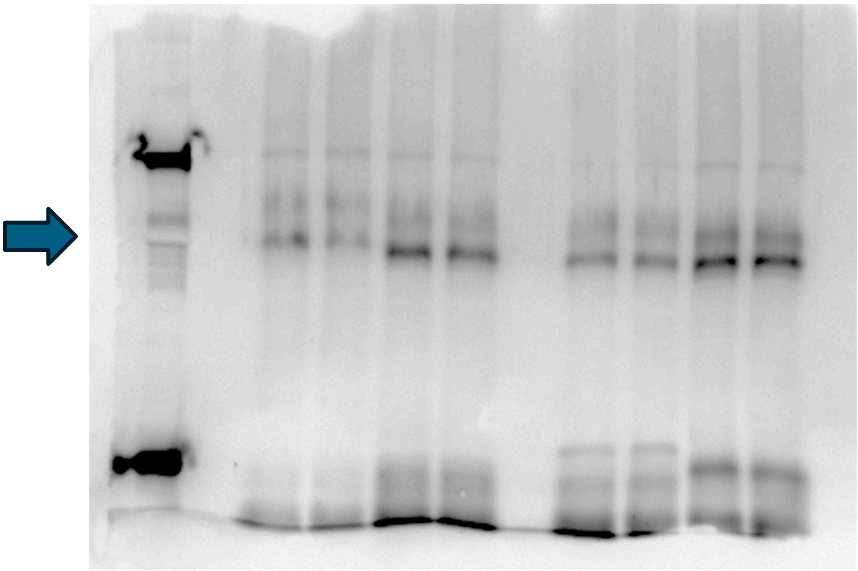

Immunoblot analysis of gut Acrolein-adducts (Fig. 3A)

Supplementary Figure S4

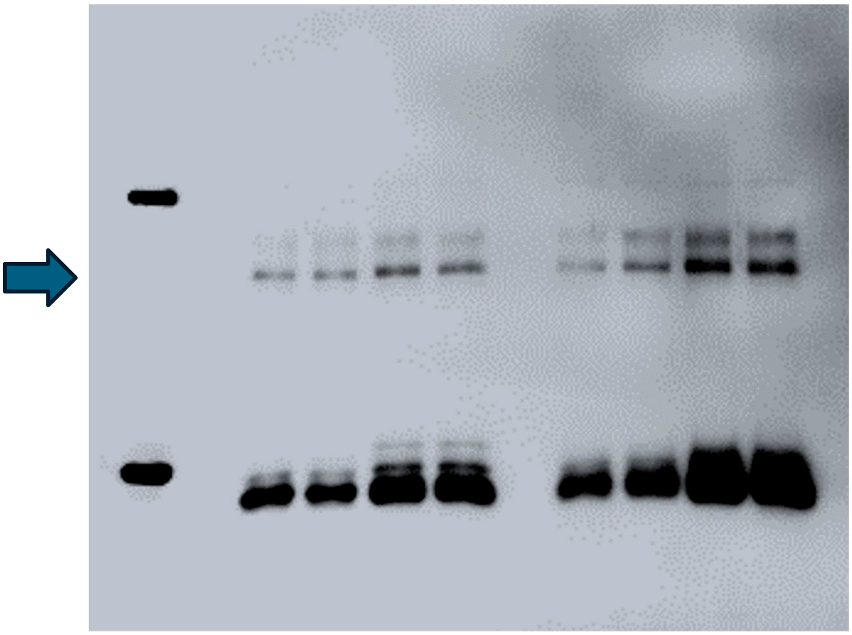

Immunoblot analysis of gut Ubiquitin-conjugates (Fig. 3A)

Supplementary Information

Supplementary Figure S5

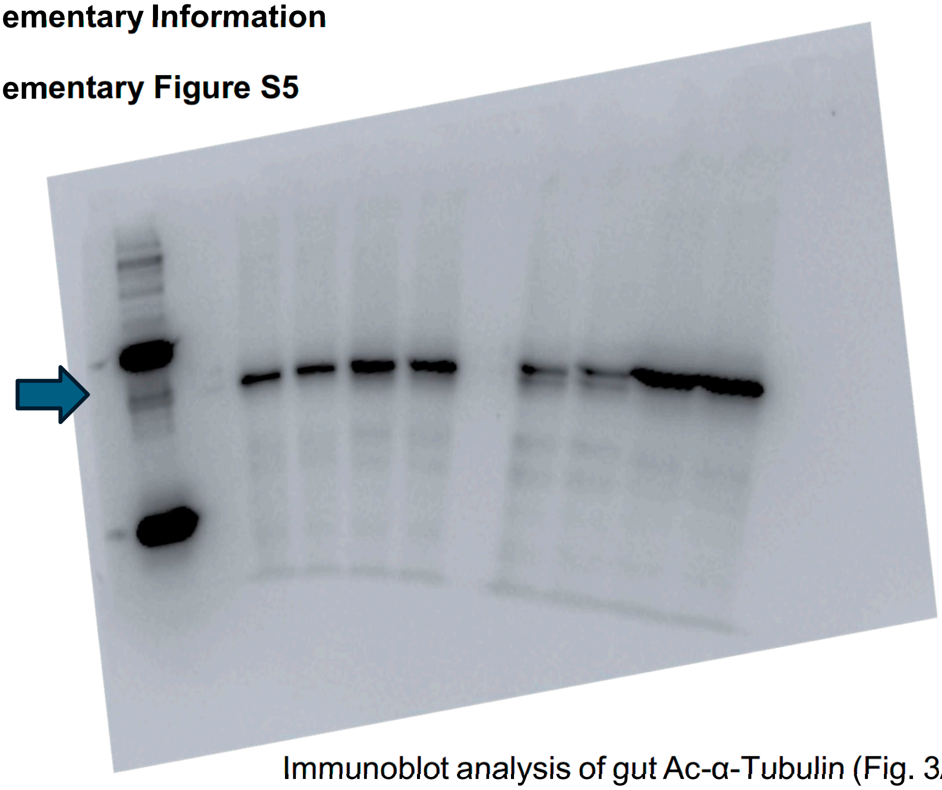

Immunoblot analysis of gut Ac-α-Tubulin (Fig. 3A)

Supplementary Figure S6

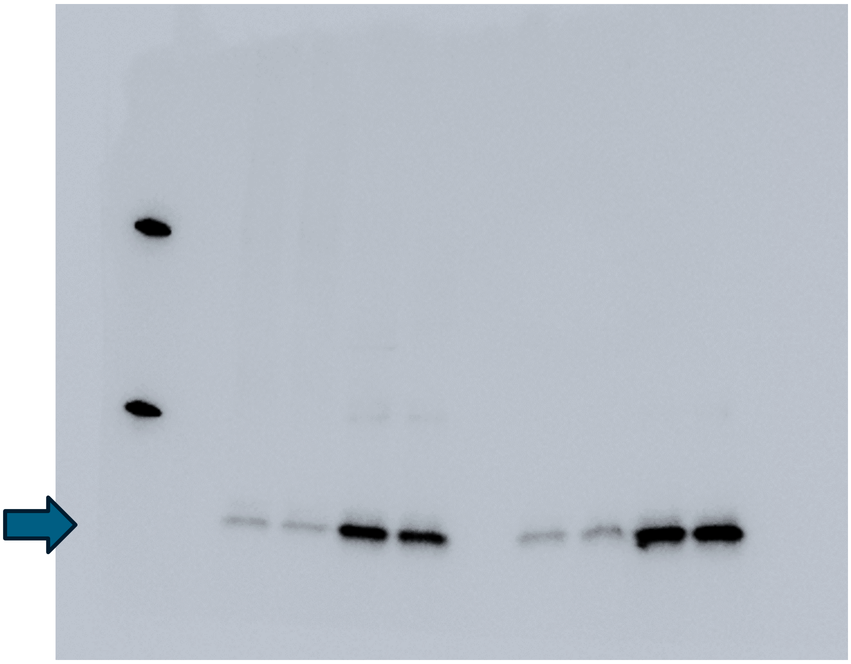

Immunoblot analysis of gut Cleaved-caspase 3 (Fig. 3A)

Supplementary Information

Supplementary Figure S7

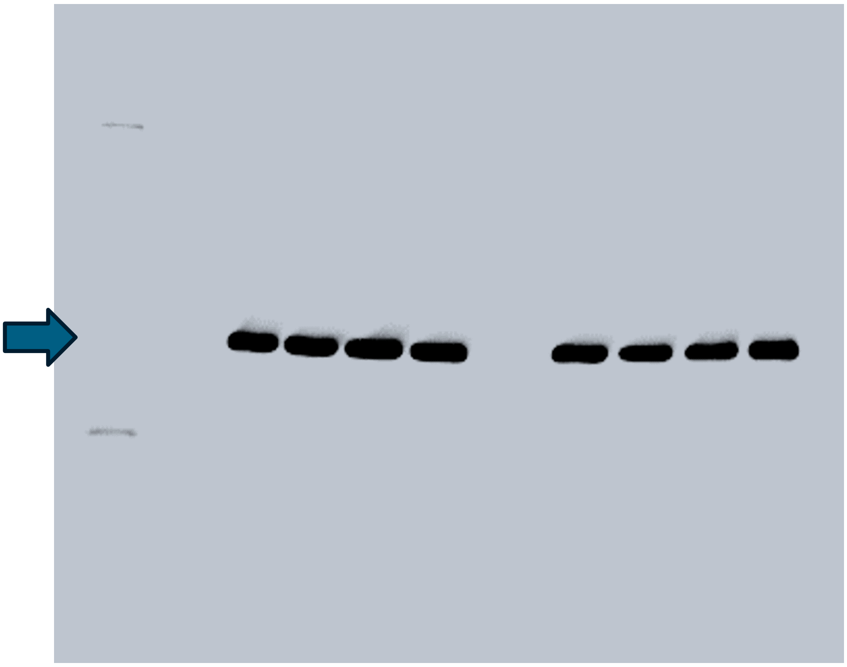

Immunoblot analysis of gut GAPDH (Fig. 3A)

Supplementary Figure S8

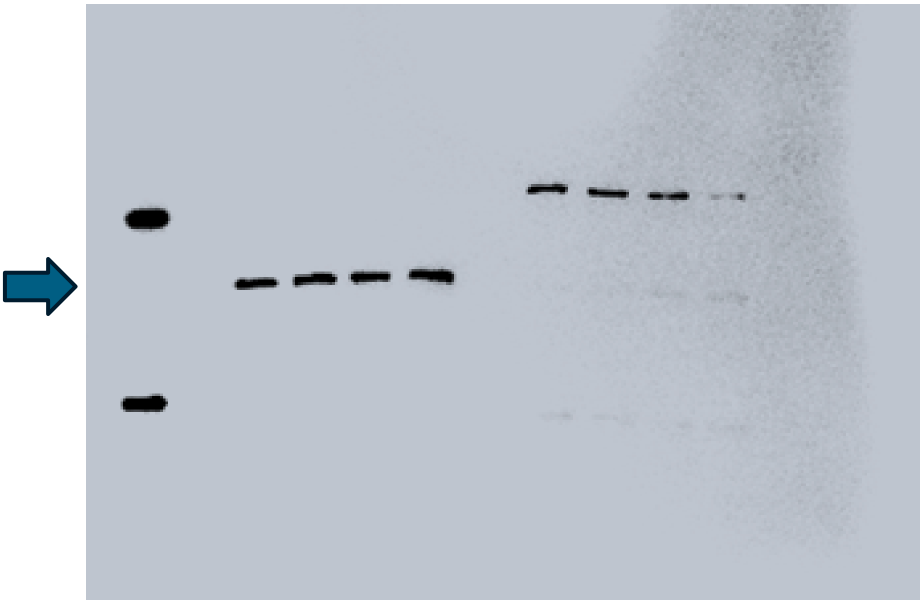

Immunoblot analysis of gut ALDH2 (Fig. 3B)

Supplementary Information

Supplementary Figure S9

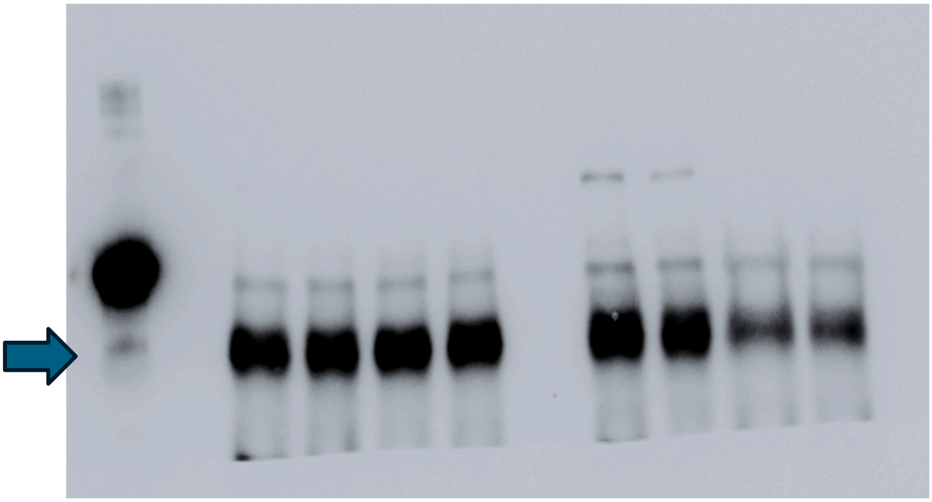

Immunoblot analysis of gut E-Cadherin (Fig. 3B)

Supplementary Figure S10

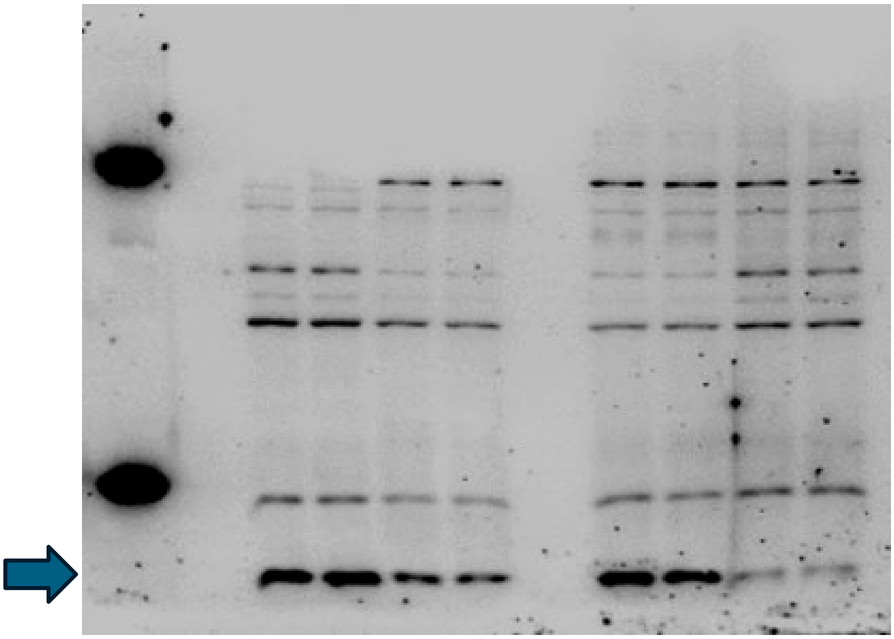

Immunoblot analysis of gut Claudin-1 (Fig. 3B)

Supplementary Information

Supplementary Figure 11

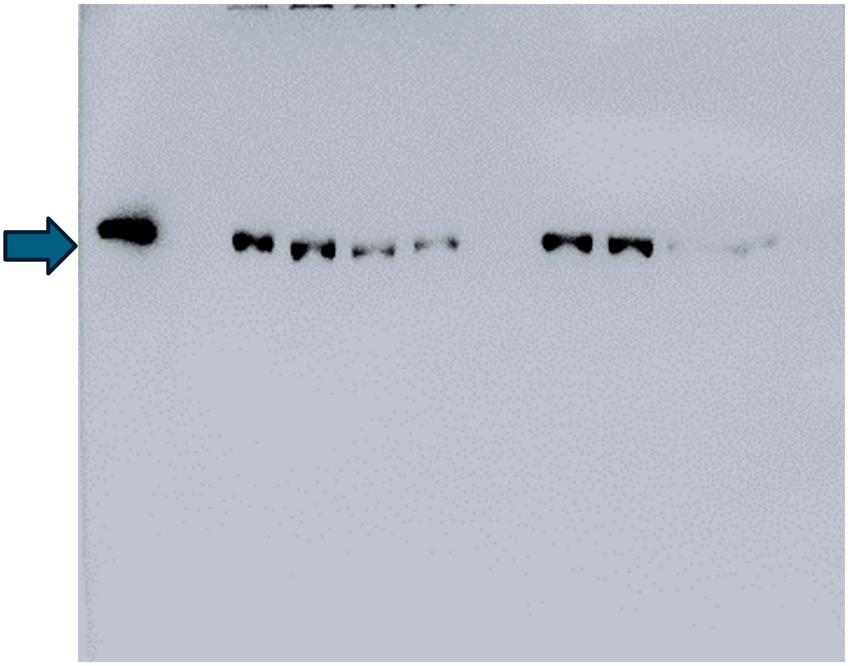

Immunoblot analysis of gut β-Catenin (Fig. 3B)

Supplementary Figure S12

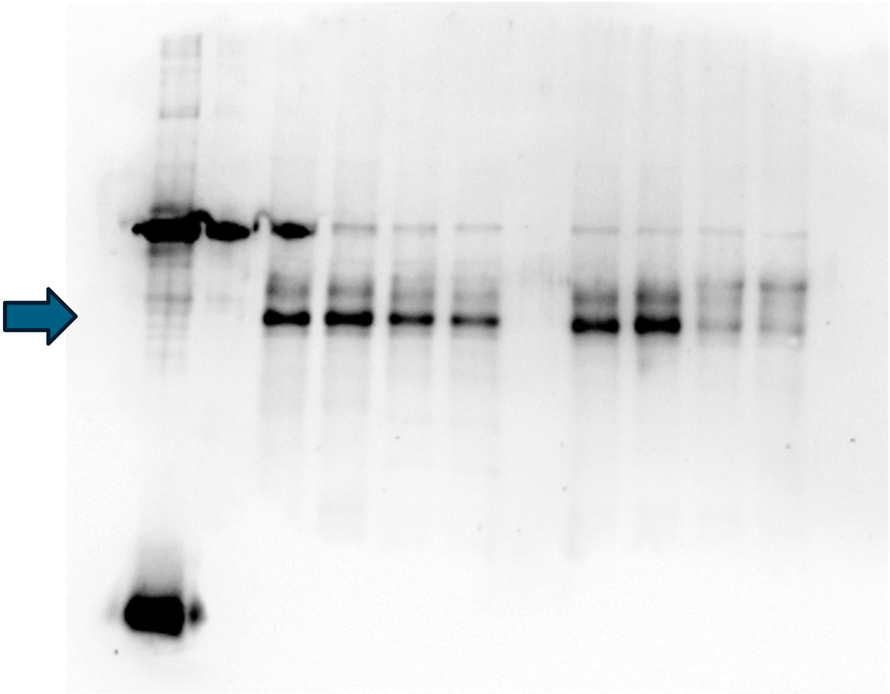

Immunoblot analysis of gut Occludin (Fig. 3B)

Supplementary Information

Supplementary Figure S13

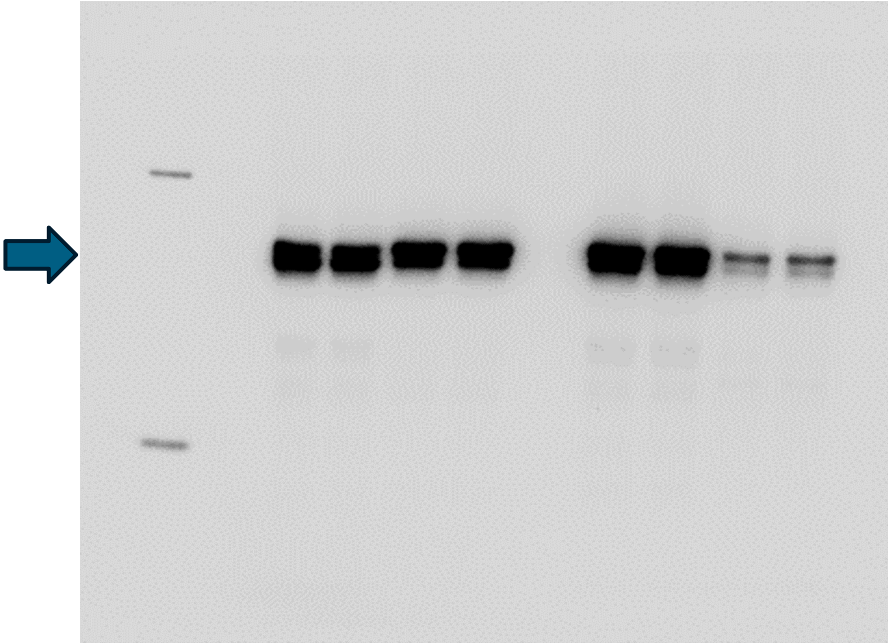

Immunoblot analysis of gut α-Tubulin (Fig. 3B)

Supplementary Figure S14

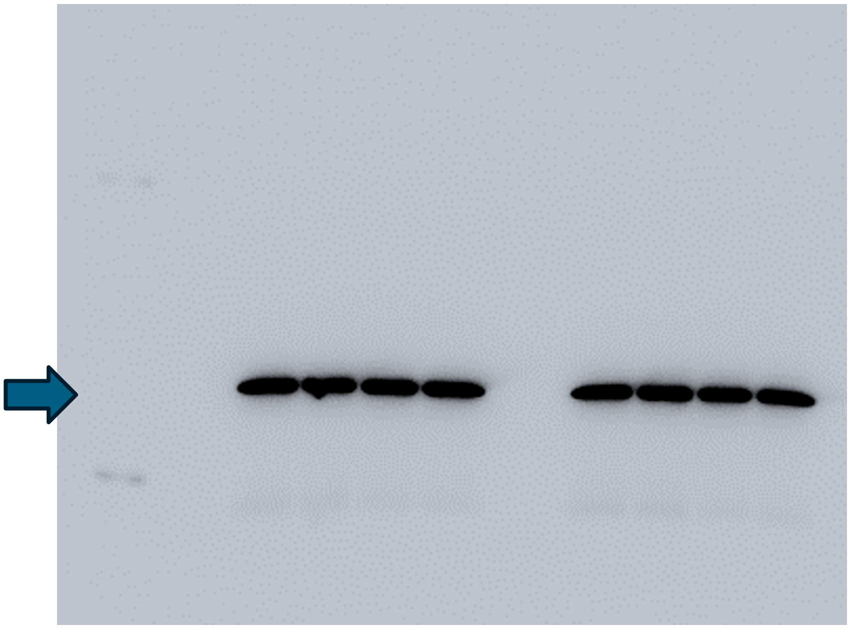

Immunoblot analysis of gut GAPDH (Fig. 3B)

Supplementary Information

Supplementary Figure S15

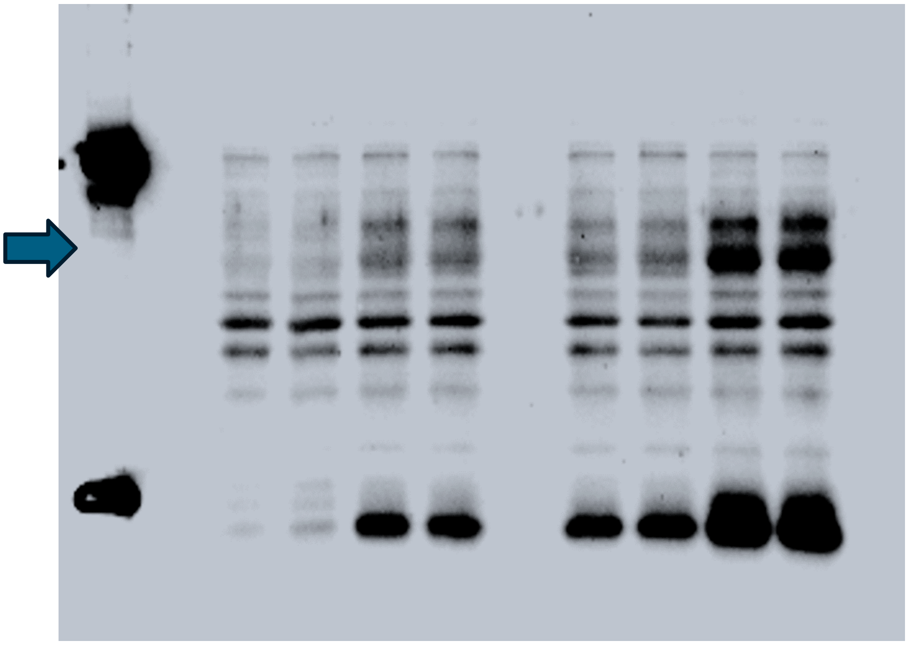

Immunoblot analysis of hippocampal Acrolein-adducts (Fig. 5)

Supplementary Figure S16

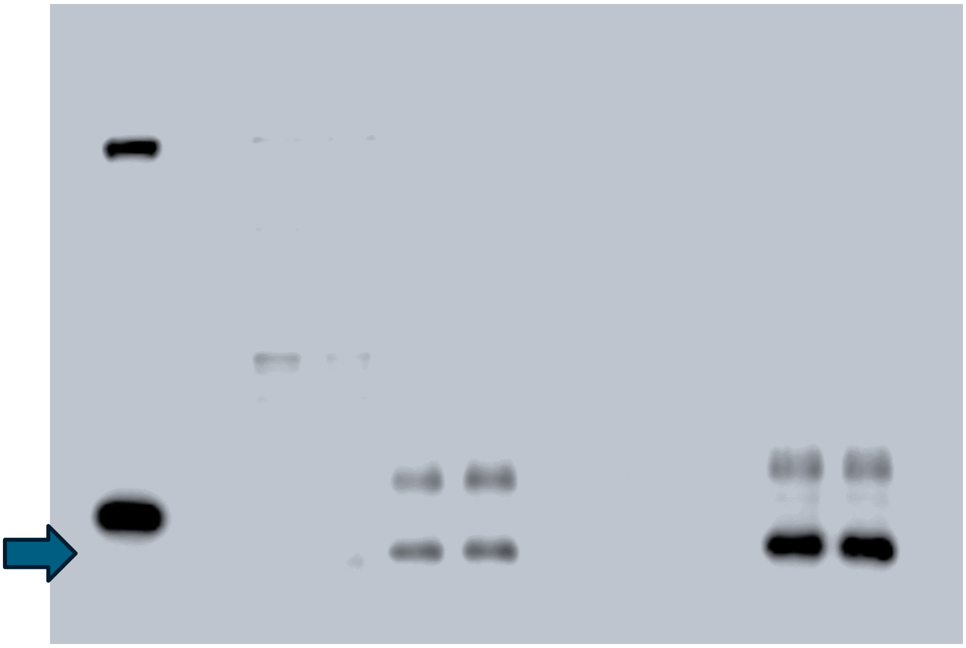

Immunoblot analysis of hippocampal Cleaved-caspase 3 (Fig. 5)

Supplementary Information

Supplementary Figure S17

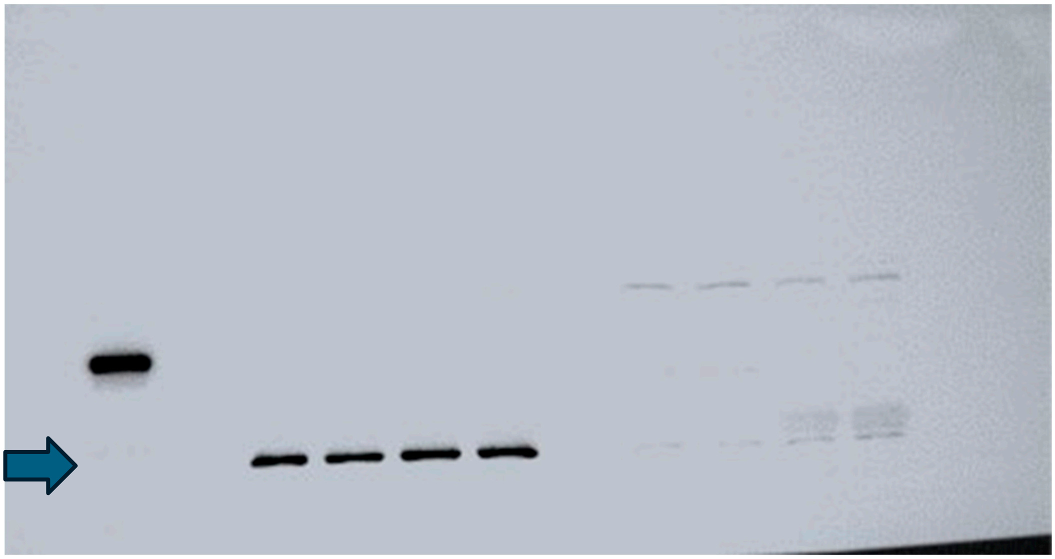

Immunoblot analysis of hippocampal ALDH2 (Fig. 5)

Supplementary Figure S18

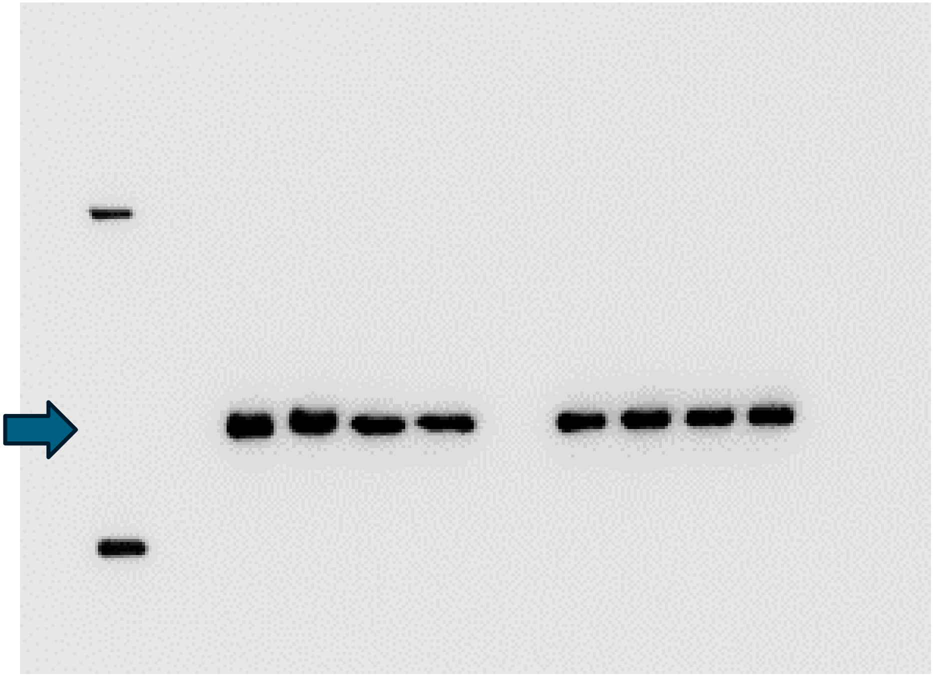

Immunoblot analysis of hippocampal GAPDH (Fig. 5)

Supplementary Information

Supplementary Figure S19

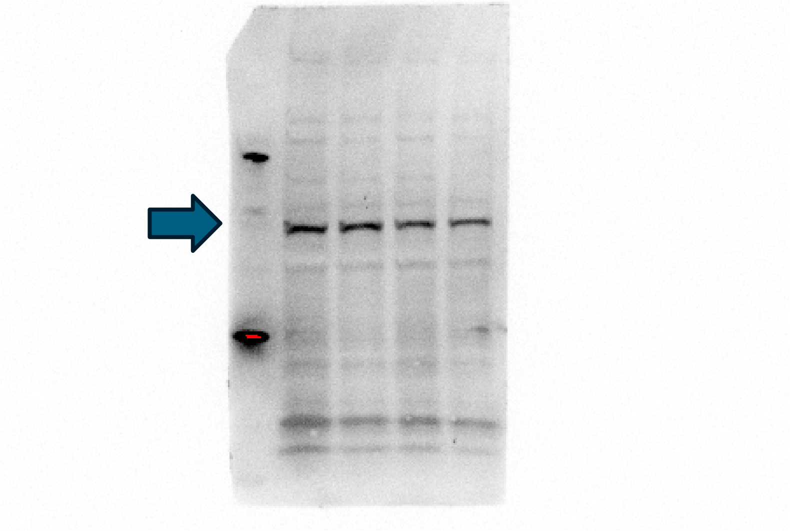

Immunoblot analysis of Neuro2A cell ALDH2 (Fig. 6B)

Supplementary Figure S20

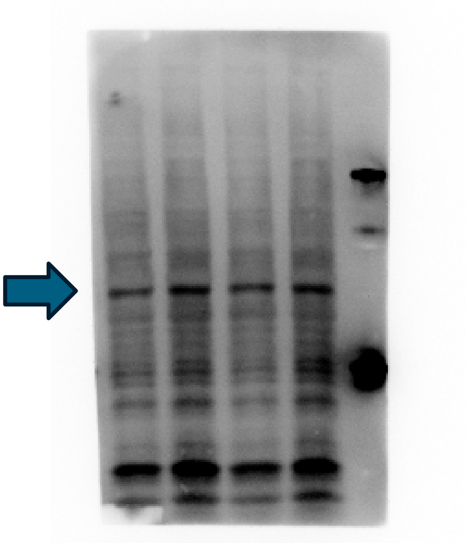

Immunoblot analysis of Neuro2A cell 3-NT (Fig. 6B)

Supplementary Information

Supplementary Figure S21

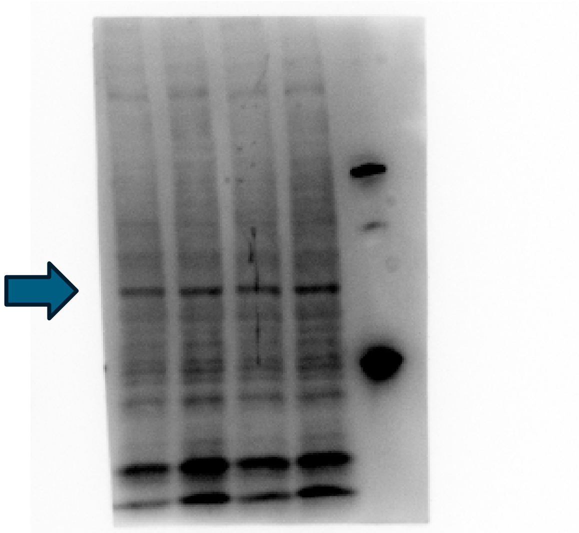

Immunoblot analysis of Neuro2A cell Ac-Lys (Fig. 6B)

Supplementary Figure S22

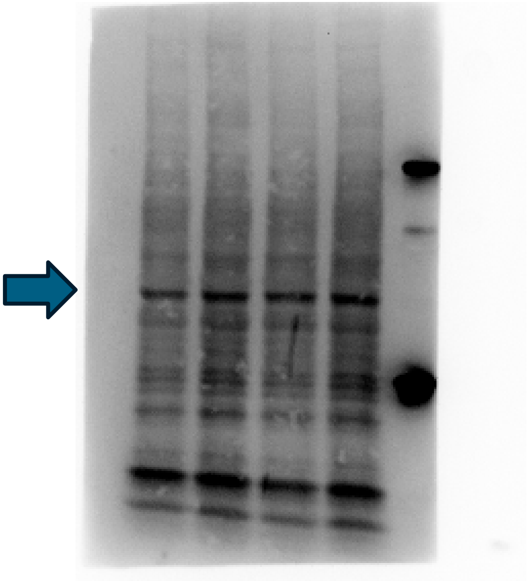

Immunoblot analysis of Neuro2A cell Acrolein-adducts ((Fig. 6B)

Supplementary Information

Supplementary Figure S23

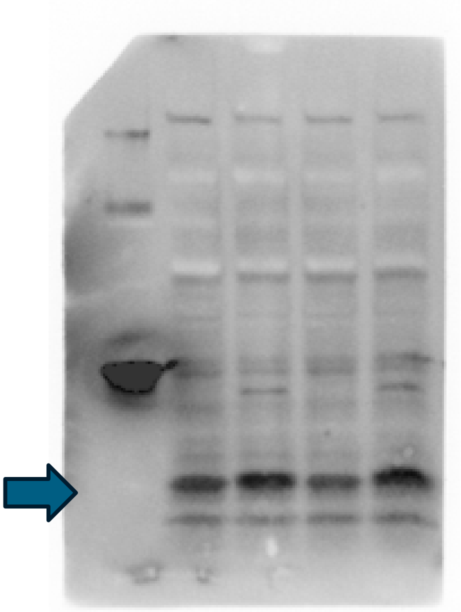

Immunoblot analysis of Neuro2A cell Cleaved-caspase 3 (Fig. 6B)

Supplementary Figure S24

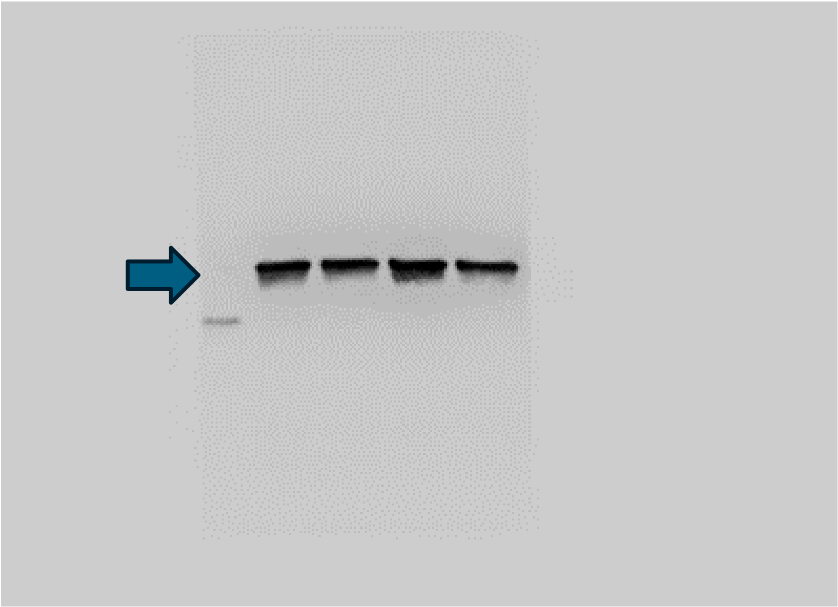

Immunoblot analysis of Neuro2A cell GAPDH (Fig. 6B)
